# ﻿*Justiciatiandengensis* (Acanthaceae), a new species from Guangxi, China

**DOI:** 10.3897/phytokeys.260.161109

**Published:** 2025-07-16

**Authors:** You Nong, Bin Feng, Qi-Min Hu, Ying-Jing Li, Gui-Yuan Wei, Xing-Yun Ji, Ke-Dao Lai

**Affiliations:** 1 Guangxi Key Laboratory of Traditional Chinese Medicine Quality Standards, Guangxi Institute of Chinese Medicine & Pharmaceutical Science, No. 20–1 Dongge Road, Nanning, Guangxi, China Guangxi Institute of Chinese Medicine & Pharmaceutical Science Nanning China; 2 State Key Laboratory of Dao-di Herbs, Beijng, 100700, China State Key Laboratory of Dao-di Herbs Beijing China

**Keywords:** *Justicia*, karst cave, new species, taxonomy

## Abstract

*Justiciatiandengensis* (Acanthaceae), a new species from a karst cave in southwestern Guangxi, China, is described and illustrated based on morphological and molecular evidence. This new species resembles *Justicialeptostachya* Hemsl. in having elongated stems, cauline leaves, and more than one flower per rachis node, but it can be readily distinguished by its oblong, thickly papery leaf blades (vs. ovate, thinly papery); calyx 2–3 mm long (vs. ca. 6 mm long); and pubescent ovary and style (vs. glabrous). Photographs, an illustration, and a distribution map are also provided.

## ﻿Introduction

*Justicia* L. is the largest genus of Acanthaceae, distributed throughout Africa, tropical and subtropical Asia, the southwest Pacific, and tropical and subtropical America. It is believed to be one of the most complex and morphologically diverse genera of the family (Graham 1988; Ezcurra 2002), now with 913 accepted names (POWO 2025). The genus is characterized by the tubular and bilabiate corolla with a stylar furrow (rugula) in the upper lip, two bithecous stamens – usually with the lower anther-thecae spurred at the base – “Knötchenpollen” pollen grains, and 4- (rarely 2-) seeded stalked capsules (Lindau 1894; Graham 1988; McDade et al. 2000; Hu et al. 2011; Deng et al. 2016; Deng 2020).

Since Graham’s pioneering work (Graham 1988), in which the author divided the genus *Justicia* into 16 sections based on morphological characters including inflorescence, androecium, pollen, and seed traits, several studies using a molecular phylogenetic approach have been conducted within *Justicia* and allied genera. These studies demonstrate the complexity of relationships among taxa and do not fully support Graham’s sections (McDade et al. 2000; Kiel et al. 2018). Recent phylogenetic studies have indicated that *Justicia* s.l. is a polyphyletic group, with its members randomly nested within other genera of the tribe Justicieae (McDade et al. 2010; Deng et al. 2016; Kiel et al. 2017, 2018). In 2023, Niu et al. (2023) published a study on the complete chloroplast genomes of 13 Asian *Justicia* (Acanthaceae) species and divided all sampled *Justicia* species in the present study into three informal clades. In Clade I, *J.grossa* C. B. Clarke (the type of Justiciasect.Grossa) is the earliest diverging species, sister to the monospecific genus *Clinacanthus* Nees. Both belong to subtribe Tetramerinae of tribe Justicieae. Clade II includes a single sampled species of *Rungia* Nees and six sampled species of *Justicia*, including *J.gendarussa* Burm. f., *J.ventricosa* Wall. ex Hook. f., *J.lianshanica* (H. S. Lo) H. S. Lo, *J.latiflora* Hemsl., *J.patentiflora* Hemsl., and *J.leptostachya* Hemsl. Clade III is sister to Clade II and contains *Peristrophejaponica* (Thunb.) Bremek., five sampled species of *Dicliptera* Juss., and nine sampled species of *Justicia*.

During our field surveys in Tiandeng County, Guangxi, in March 2025, we discovered a distinct *Justicia* population in a karst cave. This population resembles *J.leptostachya* in having elongated stems, cauline leaves, and more than one flower per rachis node. However, it differs notably by its pubescent ovary and style. After consulting the relevant literature (Deng et al. 2016; Tong and Deng 2019, 2021) and examining related specimens, we confirm that this unusual plant represents a new *Justicia* species. Herewith, the new species is described and illustrated in detail.

## ﻿Materials and methods

### ﻿Morphology

The new species was described based on field observations made in March and April 2025 and examination of herbarium specimens at GXMI. Other related *Justicia* species were examined using online images from the Kew Herbarium Catalogue (http://apps.kew.org/herbcat/gotoHomePage.do) and JSTOR Global Plants (http://plants.jstor.org/). Morphological characters that distinguish it from all other species in the genus *Justicia* were used. We also observed living plants of the new species at flowering and fruiting time (March and April), focusing on characters of the stems, petioles, leaf blades, inflorescences, bracts and bracteoles, calyces, corollas, staminal filaments, ovaries and styles, and capsules.

Descriptions were written based on herbarium specimens. Measurements were taken using a tape measure and calipers. The structure and distribution of the indumentum were observed and described under a dissecting microscope at magnifications greater than 20×. Additional information on locality, habitat, ecology, plant form, and fruits was collected in the field and from herbarium labels. The conservation threat assessment followed the IUCN Categories and Criteria (IUCN 2022).

### ﻿Molecular phylogenetic analysis

Leaf material of the putative new species was collected and stored with silica gel in zip-lock plastic bags until use for comparisons and taxonomic treatment. In this study, molecular phylogenetic analysis based on the ITS dataset was first conducted to resolve the phylogenetic position of the new species. Genomic DNA of the potential new species was extracted from silica-gel-dried leaves using the modified 2× CTAB procedure of Doyle and Doyle (1987). Primers used for polymerase chain reaction (PCR) amplification and sequencing were the same as those used by Chen et al. (2021), while PCR procedures followed those described in Chen et al. (2016). Another 23 sample sequences were obtained from NCBI. The specimen information and GenBank accession numbers for all sequences are listed in Table 1.

**Table 1.** Vouchers of specimens and GenBank accession numbers.

Accession no.	Taxon	Voucher
NC_080243.1	*Justicialianshanica*	Hezhou, Guangxi, Li J.L. IBK00434308 (IBK)
NC_080238.1	*Justiciapatentiflora*	Puer, Yunnan, Liu E.D. 1224673 (KUN)
NC_044668.1	*Justicialeptostachya*	Longzhou, Guangxi, Wang H. 01558012 (PE)
NC_080236.1	*Justicialatiflora*	Wulong, Chongqing, Liu Z.Y. 01861907 (PE)
GQ436497.1	*Justiciaventricosa*	Mengla, Yunnan, Zhou S.S. 01558037 (PE)
GQ436500.1	*Justiciagendarussa*	Mengla, Yunnan, Zhou S.S. 01558008 (PE)
NC_047476.1	*Justiciaadhatoda*	Fangchenggang, Guangxi, Huang Y. S. IBK00220366 (IBK)
NC_044862.1	*Justiciaflava*	Kenya, Guo Y. J. 1385954 (KUN)
NC_080235.1	*Justiciabetonica*	Hongkong, S.Y. Hu & K.H.Yung 01545987 (PE)
NC_080239.1	*Justiciavagabunda*	Mengla, Yunnan, Guo Y.J. 1385987 (KUN)
GQ436501.1	*Clinacanthusnutans*	Mengla, Yunnan, Zhou S.S. 01545415 (PE)
NC_080240.1	*Justiciagrossa*	–
MF963219.1	*Justiciacalifornica*	American, Li B.S. 01806354 (PE)
NC_080234.1	*Justiciaquadrifaria*	Lushan, Jiangxi, Liang T.J. 02042929 (PE)
L01930.2	*Justiciaodora*	–
NC_080237.1	*Justiciamollissima*	Yunnan, M.Iabbe Delavay 1220804 (KUN)
NC_080242.1	*Justiciademissa*	–
MT233545.1	*Justicialepida*	–
MH356484.1	*Justiciacarnea*	Hongkong, K.Y. CHAN 01545998 (PE)
MN848245.1	*Justiciaprocumbens*	Napo, Guangxi, Qin H.N. 02011426 (PE)
L14401.1	*Justiciaamericana*	American, Loy R.Phillippe 02002878 (PE)
KJ773606.1	*Justiciaovata*	American, M.T.Hall 01558014 (PE)
ON951194.1	*Justicianyassana*	–
–	*Justiciatiandengensis*	Tiandeng, Guangxi, 051210 (GXMI)

All sequences were assembled and edited using Geneious v.7.06 (Kearse et al. 2012), then aligned using MUSCLE (Edgar 2004) and manually adjusted in MEGA 6.0 (Tamura et al. 2013). Bayesian inference (BI) (Ronquist et al. 2012) and maximum likelihood (ML) (Stamatakis 2014) analyses were used for phylogenetic reconstruction, and settings for both analyses followed those described by Chen et al. (2021).

The optimal model was determined using ModelFinder (Kalyaanamoorthy et al. 2017) based on the Bayesian Information Criterion (BIC). Maximum likelihood (ML) analysis was conducted using IQ-TREE 2.1.3 (Nguyen et al. 2015) with 1,000 ultrafast bootstrap replicates under the K80 model identified by ModelFinder. Bayesian inference (BI) analysis was implemented in MrBayes 3.2.6 using the K80 model, with two independent runs of 1,000,000 generations each, sampling every 1,000 generations. Convergence was assessed based on the average standard deviation of split frequencies (< 0.01) and effective sample size (> 200). The top 25% of sampled trees were discarded as burn-in, and posterior probabilities (PP) were calculated from the remaining trees. Branch support values were considered statistically significant when ML bootstrap support (MLBS) values were ≥ 70% (Huelsenbeck and Hillis 1993) and posterior probabilities were ≥ 0.95 (Leaché and Reeder 2002). After excluding taxa showing strong conflict between the nuclear and plastid trees, the combined nuclear and plastid datasets were concatenated for phylogenetic analyses. *Clinacanthusnutans* (Burm. f.) Lindau was used as the outgroup.

## ﻿Results and discussion

**Figure 1.**
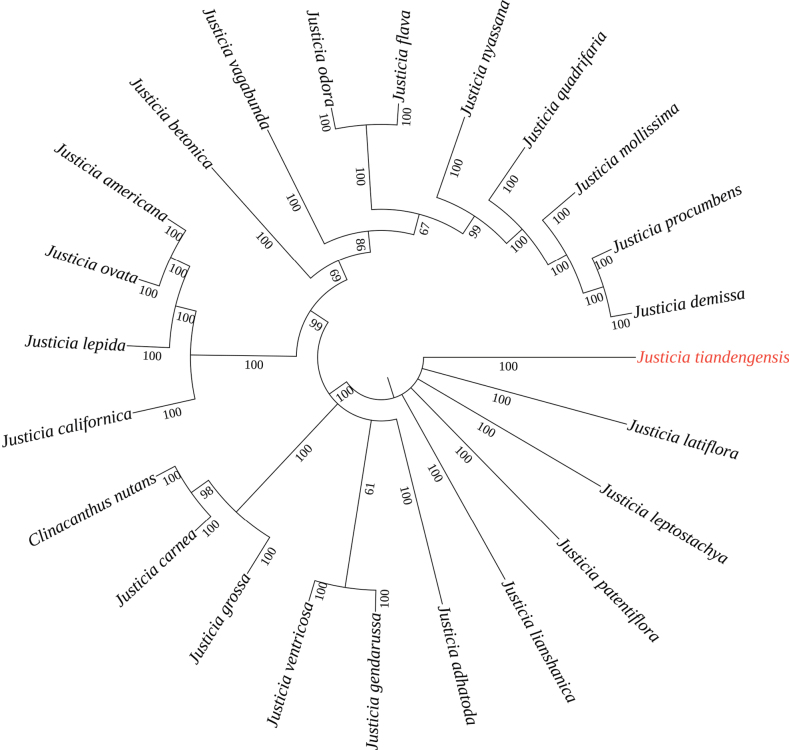
ML tree of the new species *Justiciatiandengensis* sp. nov. and its related species, based on the ITS dataset. Bootstrap values of the maximum likelihood are shown along the branches.

The ITS dataset comprises 24 accessions representing 24 species, including the outgroup (Table 1). The aligned matrix of ITS sequences was 722 bp in total. The ML result is shown in Fig. 1. The samples of the putative new species (indicated with a red background) clustered into a strongly supported monophyletic lineage. Based on morphological characters and phylogenetic results, we recognize this unfamiliar plant as a distinct species and describe it here as *Justiciatiandengensis* Y. Nong & G. Y. Wei.

### ﻿Taxonomic treatment

#### ﻿ Justicia tiandengensis

Taxon classificationPlantaeLamialesAcanthaceae

Y.Nong & G.Y.Wei sp. nov.

116F4670-37E0-58F4-8487-3AB17114484B

urn:lsid:ipni.org:names:77365659-1

**Figure 2.**
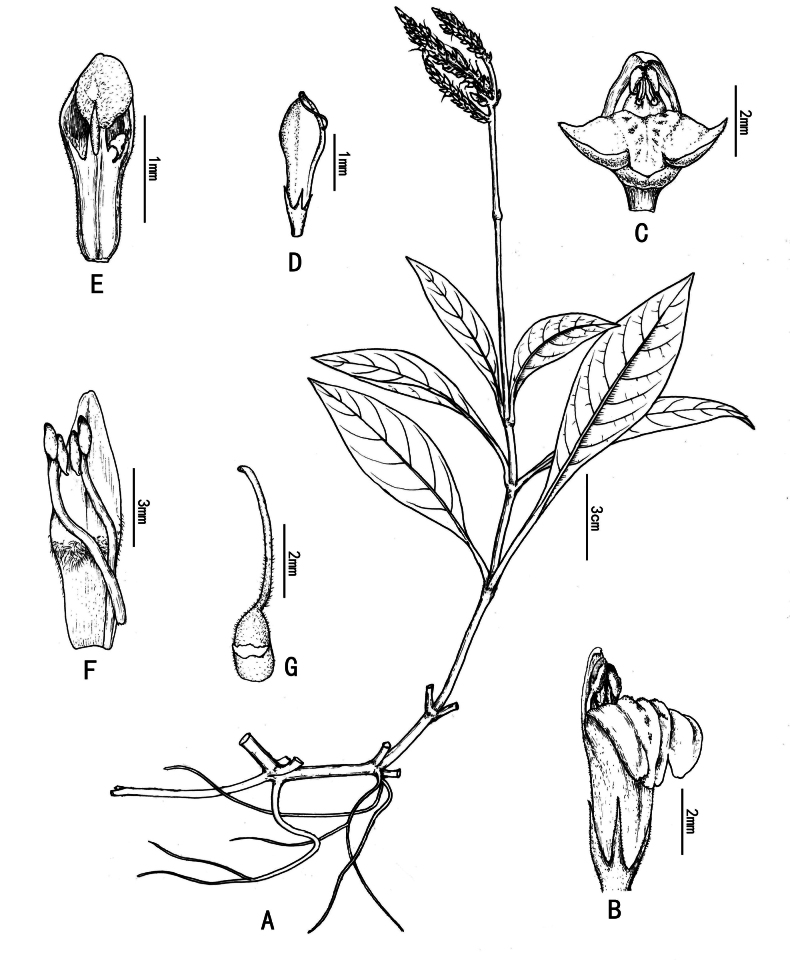
Line drawing of *Justiciatiandengensis* Y.Nong & G.Y. Wei. **A.** Flowering plant; **B.** Flower (lateral view); **C.** Flower (front view); **D.** Capsule; **E.** Capsule dissection; **F.** Flower dissection (showing lower anther thecae spurred at base); **G.** Ovary and style (drawn by Xin-cheng Qu).

**Figure 3.**
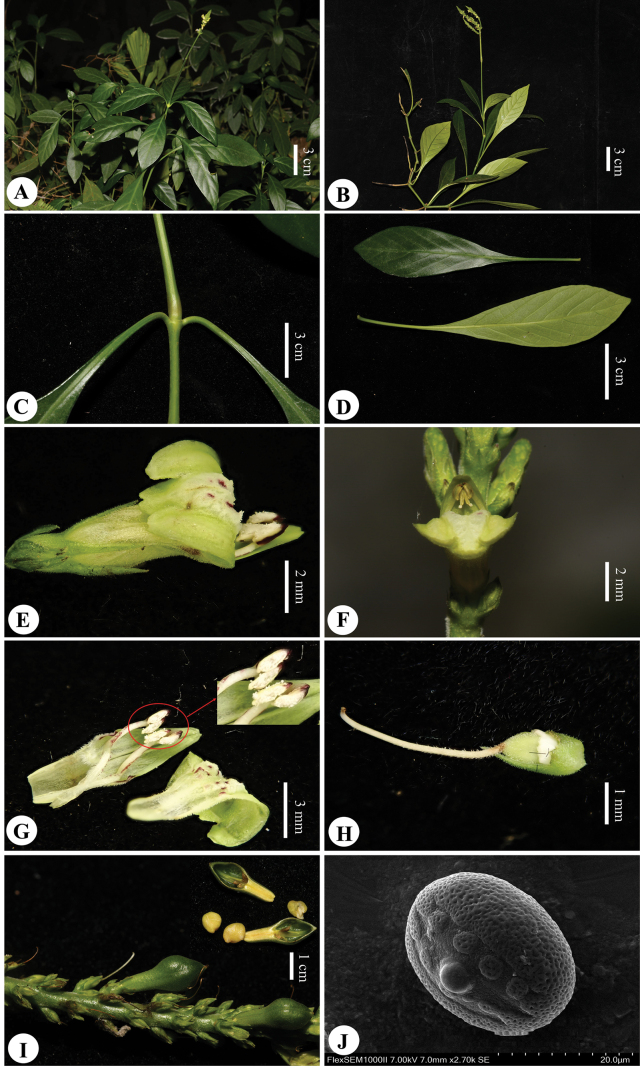
*Justiciatiandengensis* Y.Nong & G.Y. Wei. **A.** Habitat; **B.** Flowering plant; **C.** Stem and node; **D.** Leaves (abaxially and adaxially); **E.** Flower (lateral view); **F.** Flower (front view, showing only lower anther theca spurred at base); **G.** Flower (dissected view); **H.** Ovary and style; **I.** Capsule and seeds; **J.** Pollen grain (Edited by Y. Nong).

**Figure 4.**
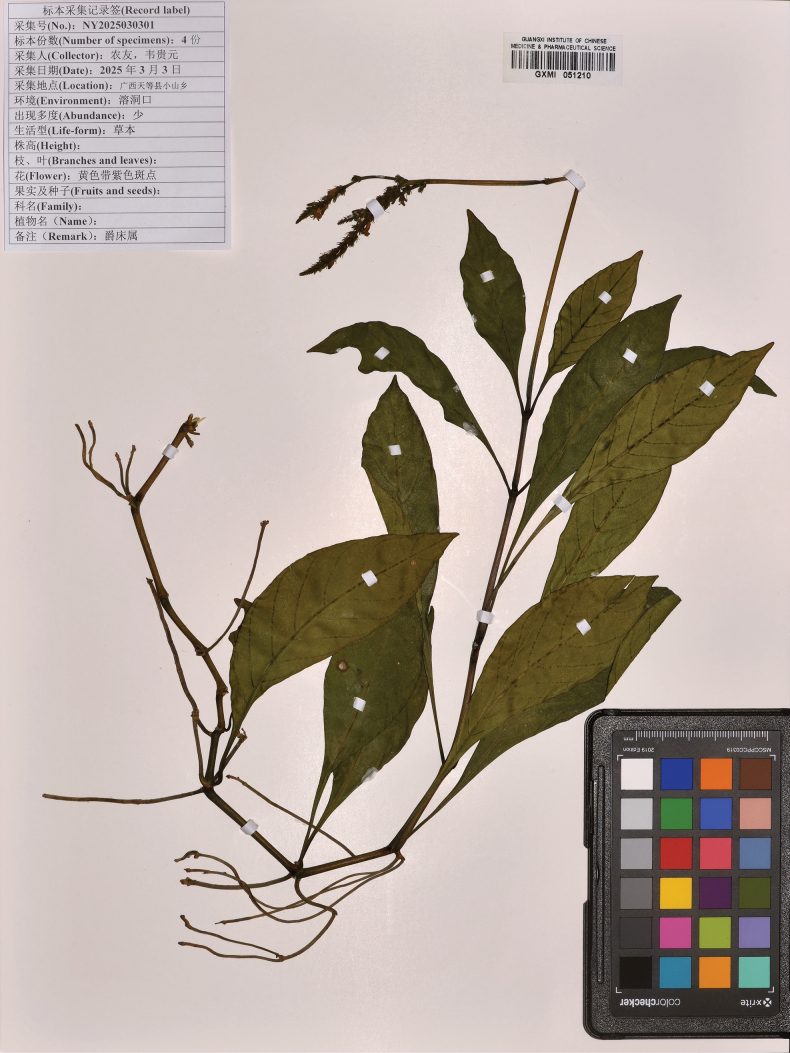
The type specimen of *Justiciatiandengensis* Y.Nong & G.Y. Wei.

Figs 1 , 2 , 3 , 4 Chinese name: “tiān děng jué chuáng” (天等爵床)

##### Diagnosis.

*Justiciatiandengensis* is most similar to *J.leptostachya*, but it can be easily distinguished by its stem terete, glabrous (vs. 4–angled, sulcate, bifariously pubescent), its leaf blade oblong, thickly papery (vs. ovate, thinly papery); its calyx 2–3 mm long, outside pubescent (vs. ca. 6 mm long, margin ciliate); and its ovary and style pubescent (vs. glabrous).

##### Type.

China - Guangxi • Y Nong et al. 051210 (GXMI); Tiandeng County; 23°57'26"N, 107°04'53"E; alt. 410 m; 3 Mar. 2025; fl • Y Nong et al. NY2025050601 (GXMI, IBK); Tiandeng County; 23°57'26"N, 107°04'53"E; alt. 410 m; 6 May 2025; fl, fr.

##### Description

. Herbs to 40 cm tall. Stems green, terete, basally prostrate and rooting at nodes, then erect, glabrous. Petiole 1–4 cm, not winged, glabrous; leaf blade oblong, 5–12 × 1.7–3.5 cm, thickly papery, both surfaces glabrous, midrib sunken adaxially and prominently raised abaxially; lateral veins are approximately 5–7 pairs, slightly raised abaxially, base cuneate, decurrent, margin entire, revolute when dry, apex acute. Inflorescences green, erect, terminal, racemose or panicle, branched or unbranched; rachis puberulent, with 2–4 flowers per node; pedicels very short, ca. 0.5 mm long; bracts ovate, elliptic, or lanceolate, green, ca. 1 mm long, abaxially puberulent; bracteoles lanceolate to rarely triangular, 0.5 mm long, abaxially puberulent. Flowers suberect. Calyx 5-lobed almost to base, narrowly linear, 2–3 mm long, green, pubescent outside, clasping the corolla tube, apex acute. Corolla yellow, 6–7 mm; lower lip ca. 4 mm, 3-lobed, lobes ovate and ca. 0.5 × 0.5 mm; upper lip subtriangular, ca. 3 mm, 2-lobed. Staminal filaments ca. 5 mm, glabrous; anther thecae linear, ca. 1 mm, lower one spurred at base. Ovary ellipsoid, ca. 1 mm, pubescent, ovules 1 or 2 per locule; style ca. 5 mm, pubescent. Capsule clavate, 10–12 mm long, puberulent, base solid, 3 or 4-seeded. Seeds broadly ovate, compressed, yellowish-brown, 2–3 mm in diameter, testa tuberculate.

##### Etymology.

The specific epithet “tiandengensis” refers to the type locality, Tiandeng County (天等县), which is situated in southwest Guangxi, southwest China.

##### Distribution and habit.

**Figure 5.**
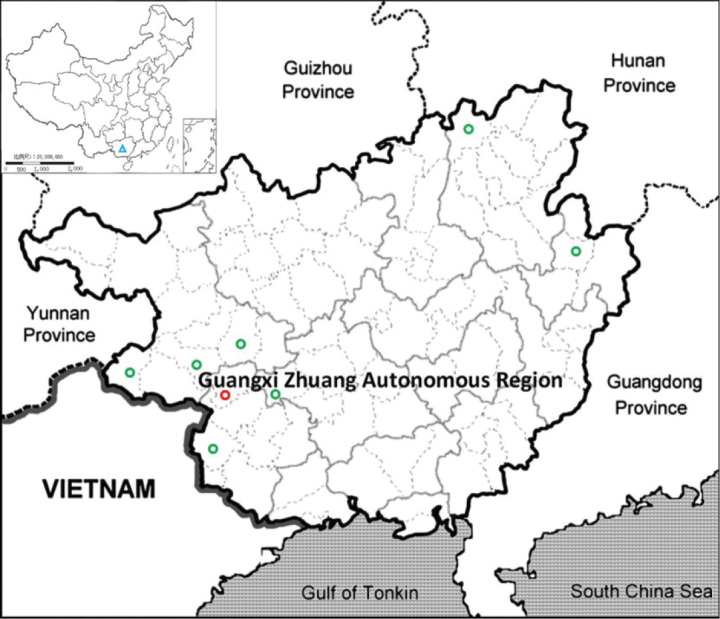
The distribution of *Justiciatiandengensis* Y.Nong & G.Y. Wei (red circle) and *J.leptostachya* Hemsl. (green circle) in Guangxi (blue triangle), China.

Currently, *Justiciatiandengensis* is known only from the southwest of Guangxi, China (Fig. 5). It has been mainly found in a karst cave at an elevation of about 410 m.

##### IUCN Red List Category.

Due to limited available data, the conservation status of this new species cannot be definitively assessed. Following IUCN criteria (IUCN 2022), it is currently classified as **Data Deficient (DD)** pending further research and information.

### ﻿Discussion

In addition, *Justiciatiandengensis* is also similar to *J.damingensis* (H. S. Lo) H. S. Lo, but it differs in having glabrous stems (vs. bifariously pubescent), glabrous petioles (vs. pubescent), and a pubescent ovary and style (vs. glabrous). More detailed morphological differences among the similar species are presented in Table 2.

**Table 2.** Main morphological differences amongst *Justiciatiandengensis*, *J.grossa*, *J.leptostachya*, and *J.damingensis*.

Morphological traits	*Justiciatiandengensis*	*J.leptostachya*	*J.damingensis*
**Habit**	Herbs	Herbs	Herbs
**Plant**	30–40 cm tall	40–60 cm tall	15–30 cm tall
**Stems**	terete, basally prostrate and rooting at nodes, then erect, glabrous	4–angled, sulcate, bifariously pubescent	subterete, basally prostrate and rooting at nodes, then erect, sulcate, bifariously pubescent
**Petiole**	1–4 cm, not winged, glabrous	1–2 cm	0.5–1.5 cm, pubescent
**leaf blade**	oblong, 5–12 × 1.7–4 cm, thickly papery, both surfaces glabrous, midrib sunken adaxially and prominently raised abaxially; lateral veins are approximately 5–7 pairs, slightly raised abaxially, base cuneate, decurrent, margin entire, revolute when dry, apex acute	ovate–lanceolate, 10.5–12.5(–18) × 5–6.5(–8) cm, papery, abaxially strigose along veins, adaxially sparsely strigose, secondary veins 5–9 on each side of midvein, base broadly cuneate, margin entire or slightly undulate, apex acuminate	ovate, 4–9 × 2.5–4.5 cm, thinly papery, abaxially pubescent along veins, adaxially glabrous, secondary veins ca. 5 on each side of midvein, base narrowly cuneate, margin shallowly undulate, apex acute
**Inflorescences**	terminal, racemose, or panicle, branched or unbranched; rachis puberulent, with 2–4 flowers per node	terminal, spikes, branched or rarely unbranched; rachis hispid, with several flowers per node	terminal, spiciform, branchless, or trifurcate branched, with 3 flowers per node
**bracts and bracteoles**	bracts ovate, 1–2 × 0.5–1 mm, abaxially puberulent; bracteoles triangular, 1–2 × 0.5–1 mm, abaxially puberulent	bracts linear, ca. 2 × 0.8 mm, abaxially puberulent; bracteoles similar to bracts	bracts subulate, 1.5–2 mm, abaxially pubescent; bracteoles similar to bracts.
**Calyx**	2–3 mm long, outside pubescent	ca. 3 mm long, outside puberulent	ca. 6 mm long, margin ciliate
**Corolla**	yellow, 6–7 mm; lower lip 3–lobed, lobes ovate; upper lip subtriangular	yellow, ca. 6 mm; lower lip patent, 3–lobed, lobes ovate; upper lip oblong	yellow, ca. 1 cm, lower lip, patent, 3–lobed, lobes ovate; upper lip subdeltoid
**Staminal filaments**	ca. 5 mm, glabrous; anther thecae linear, ca. 1 mm, lower one spurred at base	ca. 3 mm, glabrous; anther thecae superposed, lower one spurred at base	ca. 3 mm, glabrous; anther thecae ellipsoid, superposed, lower one spurred at base
**Ovary and style**	ovary pubescent; style pubescent	ovary glabrous; style glabrous	ovary glabrous; style glabrous
**Capsule**	clavate, 10–12 mm long, puberulent, base solid, 3 or 4-seeded. Seeds broadly ovate, compressed, yellowish-brown, 2–3 mm in diameter, testa tuberculate.	clavate, ca. 1.2 cm, puberulent, 4–seeded	Capsule not seen

*Justiciatiandengensis* has elongated, simple, or rarely branched terminal spikes; narrow bracts subtending small flowers or clusters of small flowers; 2-colporate pollen grains; and rugulose seeds. The species also bears fruits in which the placenta separates from the capsule wall but remains attached at the apices, causing them to rise up at dehiscence. According to the study by Niu et al. (2023), it belongs to subtribe Justiciinae of tribe Justicieae. To fully elucidate the phylogenetic relationships of *Justicia*, it is necessary to integrate further analyses using genetic resources and morphological evidence from a broader sampling of *Justicia* species.

## Supplementary Material

XML Treatment for Justicia tiandengensis

## ﻿Additional information

### Use of AI

No use of AI was reported.

### Author ORCIDs

You Nong https://orcid.org/0000-0001-7004-0946

Bin Feng https://orcid.org/0000-0003-0387-3635

Qi-Min Hu https://orcid.org/0009-0003-0490-3557

Ying-Jing Li https://orcid.org/0009-0008-4849-2426

Gui-Yuan Wei https://orcid.org/0000-0003-0652-1213

Xing-Yun Ji https://orcid.org/0009-0002-4907-6850

### Data availability

All of the data that support the findings of this study are available in the main text.
